# Deciphering the molecular adaptation of the king scallop (*Pecten maximus*) to heat stress using transcriptomics and proteomics

**DOI:** 10.1186/s12864-015-2132-x

**Published:** 2015-11-23

**Authors:** Sébastien Artigaud, Joëlle Richard, Michael AS Thorne, Romain Lavaud, Jonathan Flye-Sainte-Marie, Fred Jean, Lloyd S. Peck, Melody S. Clark, Vianney Pichereau

**Affiliations:** Laboratoire des Sciences de l’Environnement Marin, LEMAR UMR 6539 CNRS/UBO/IRD/Ifremer, Université de Brest (UBO), Institut Universitaire Européen de la Mer, Plouzané, 29280 France; British Antarctic Survey, Natural Environment Research Council, High Cross, Madingley Road, Cambridge, CB3 0ET UK

**Keywords:** Marine biology, Metabolism, DNA repair, Transcription regulation, Apoptosis, Energy reserves

## Abstract

**Background:**

The capacity of marine species to survive chronic heat stress underpins their ability to survive warming oceans as a result of climate change. In this study RNA-Seq and 2-DE proteomics were employed to decipher the molecular response of the sub-tidal bivalve *Pecten maximus*, to elevated temperatures.

**Results:**

Individuals were maintained at three different temperatures (15, 21 and 25 °C) for 56 days, representing control conditions, maximum environmental temperature and extreme warming, with individuals sampled at seven time points. The scallops thrived at 21 °C, but suffered a reduction in condition at 25 °C. RNA-Seq analyses produced 26,064 assembled contigs, of which 531 were differentially expressed, with putative annotation assigned to 177 transcripts. The proteomic approach identified 24 differentially expressed proteins, with nine identified by mass spectrometry. Network analysis of these results indicated a pivotal role for GAPDH and AP-1 signalling pathways. Data also suggested a remodelling of the cell structure, as revealed by the differential expression of genes involved in the cytoskeleton and cell membrane and a reduction in DNA repair. They also indicated the diversion of energetic metabolism towards the mobilization of lipid energy reserves to fuel the increased metabolic rate at the higher temperature.

**Conclusions:**

This work provides preliminary insights into the response of *P. maximus* to chronic heat stress and provides a basis for future studies examining the tipping points and energetic trade-offs of scallop culture in warming oceans.

**Electronic supplementary material:**

The online version of this article (doi:10.1186/s12864-015-2132-x) contains supplementary material, which is available to authorized users.

## Background

Near-shore marine coastal environments are experiencing considerable changes in temperature and oxygen availability due to anthropomorphic influences, which has triggered considerable modifications in biological communities [1]. Therefore, the ability of marine species and populations to cope with environmental change underpins the maintenance of current marine ecosystems. In this respect, temperature is acknowledged as one of the most important environmental threats affecting marine organisms [2, 3]. Indeed in multiple stressor experiments investigating the interacting effects of temperature with, for example, lowered pH, the dominant effect has been identified as temperature [4–6]. Therefore understanding marine organisms’ responses to thermal acclimation and/or capacity for adaptation is a critical challenge in understanding their ecology and predicting their future distributions. For society, this is of particular interest for economically important aquaculture and fished species, such as *Pecten maximus* used in this study.

Whilst the molecular mechanisms underlying cellular responses to thermal stress have been characterised in a number of model species [7–11], the responses of marine species have largely been evaluated using a limited number of evolutionary conserved stress-responsive genes. Prime examples of these are the heat shock proteins (HSPs), particularly the inducible 70 kDa form (HSP70). Most of these studies on bivalve molluscs have been very short-term acute thermal shocks. For example, Ivanina et al. [12] showed the overexpression of selected HSPs and metallothionein (MT) in *Crassostrea virginica* exposed for 1 h at 40 °C, and Brun et al. [13, 14] characterized the heat shock response and the acquisition of thermotolerance in selected Pectinidae (*Argopecten irradians*, *Placopecten magellanicus*), and the interplay between hypoxia and temperature stress was recently characterized in *P. maximus* [15, 16]. Lang et al. [17] used a microarray containing 1675 ESTs from *Crassostrea gigas* and *C. virginica* to characterize the transcriptomic response of different families of the Pacific oyster showing contrasting degrees of thermotolerance. Of note, they showed differential expression of genes encoding HSPs, and genes involved in lipid metabolism, protection against bacterial infections and cell structural elements (e.g. collagen) in response to an acute thermal stress. Finally, using a proteomics approach to identify differences in the thermal resilience of the mussel congeners *Mytilus galloprovincialis* and *Mytilus trossulus,* HSPs and proteins combating reactive oxygen species were identified in response to a 1 h thermal challenge [18].

Whilst studies examining short exposure to acute stress can provide interesting insights into affected pathways, they may not reflect the response to a permanent gradual shift such as the predicted increase in sea surface temperature [19]. Short- and long-term exposures can produce very different responses in genes expression profiles [20]. For example, in a recent study, the effect of both acute- (within a day) and long-term- (up to 14 days) exposure to heat stress on the gene expression of *Chlamys farreri* was studied [21]. Although, the question remains as to how long is a long-term challenge. In a study of thermal stress in *C. gigas*, a Q-PCR time course showed a graduated response in the gene expression profiles with a rapid increase at 3–7 days, a decrease at 14 days, which reduced further by days 17–24. By then, expression levels were presumed to have stabilised, and the oysters acclimated to the new temperature [22]. The only other recent study dealing with long-term adaptation to thermal challenge concerned *C. gigas* (3 months, 24 °C) [4]. This study showed the essential role of lipid mobilisation, the mTOR regulatory pathway, and ultimately the induction of apoptosis as a result of chronic elevated heat stress.

Studying organisms’ adaptation to changing environments is a real challenge in the field of ecological genomics. In particular, discovery-led transcriptomic and proteomic characterizations of the responses of organisms to environmental changes offer an opportunity to understand the underlying molecular basis for adaptation. Transcriptomic and proteomic approaches are highly complementary. NGS transcriptomic tools can provide extensive catalogues of genes, even for non-model species and essential reference data for the identification of proteins. Protein production is dependent on the efficiency of transcription and translation of a gene, with as final product, the result of a variety of post-translational modifications, such as phosphorylation. Hence the level of gene expression is not always directly correlated to that of its respective proteins, proteomics is therefore closer to phenotypes than transcriptomics. However, 2-DE based proteomics is naturally biased towards highly abundant proteins, and is often limited by the availability of genomic data, especially for non-model organisms. As a consequence, in spite of its physiological relevance, proteomic strategies classically provide much less data than transcriptomics.

In this study, we used the economically important king scallop *Pecten maximus*, as a model to understand marine species’ response to thermal stress. *P. maximus* is naturally distributed along a large East Atlantic latitudinal gradient (from 31 °N to 69 °N) and lives in the subtidal zone down to a depth of 500 m (www.fao.org). It is an economically important species in the UK, France and Spain, where it is considered as a high value product [23]. In this paper, we describe the first genome-wide transcriptome analysis in the king scallop, sampled over a 56-day time course whilst subjected to a thermal challenge at three different temperatures. Coupling both transcriptomic- and proteomic- approaches, provides a complementary system biology view of heat adaptation/acclimation in this species.

## Results

### Culture conditions

**Fig. 1:**
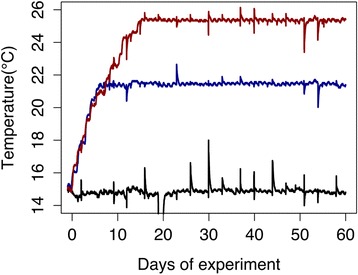
Temperatures in the three tanks during the experiment

These remained relatively stable throughout the 56 days of the experiment. The three temperatures were maintained at: 15.1 ± 0.2 °C as control, 21.4 ± 0.2 °C and 25.2 ± 0.9 °C (see Fig. 1). Salinity, pH and O_2_ were maintained at 35.8‰ ± 0.2, 8.1 ± 0.1 and 94 ± 7 %, respectively. There were slight changes to ammonia (that increased from 4.68 μM ± 1.6 to 5.96 μM ± 1.9 and 6.5 μM ± 2.3 at 21 and 25 °C, respectively).

### Physiological response to heat stress

**Fig. 2:**
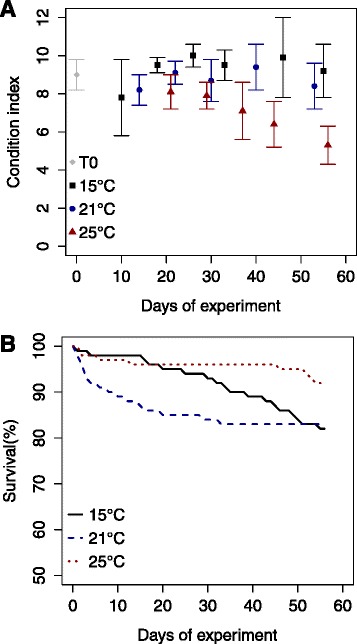
Condition index (**a**) and survival (**b**) of *Pecten maximus* during a long-term exposure (56 days) to three different temperatures (15, 21 and 25 °C)

All the scallops used in this study came from the same 2010 production of the Tinduff hatchery, and were held under identical conditions prior the start of the experiment. Hence the animals were similar in size, age and physiological condition. Mortalities occurred in each tank during the experiment (Fig. 2). However, this mortality was not correlated to the increased temperature and more than 80 % of the animals survived in all conditions (Fig. 2b). In contrast, the condition index (CI), which reflected the physiological condition of the animals, was significantly reduced at the end of the experiment for the animals kept at 25 °C (Fig. 2a).

**Fig. 3:**
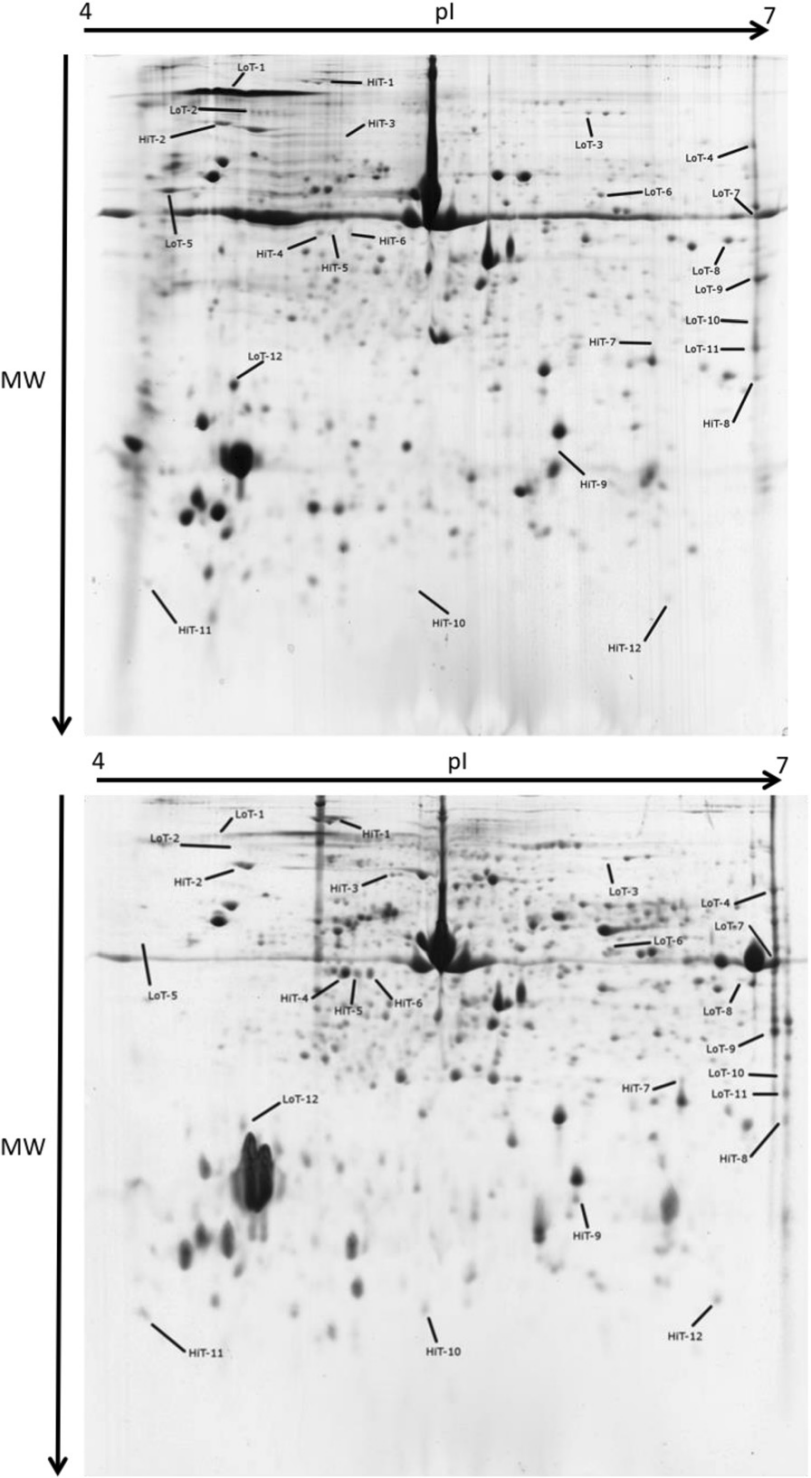
Representative bi-dimensional gels (pH 4–7, SDS-PAGE 12 %) for *Pecten maximus* mantle proteins maintained at 15 °C (*upper gel*) and 25 °C (*lower gel*). Differentially expressed spots are arrowed

### Differential gene expression analyses

RNA-Seq of the 22 different transcriptome libraries resulted in a total of 806 million paired end raw reads, which was reduced to 667 million paired end reads after quality clipping. These were assembled into a backbone assembly of 26,064 contigs with an average length of 1011 bp and a median length of 815 bp (fully described in [24]). As the aim of this study was to understand the molecular mechanisms underlying adaptation to long-term exposure to heat stress in *Pecten maximus,* a very stringent strategy was adopted to identify the consistently differentially expressed transcripts. This was performed, by taking into account those displaying a ratio (treatment/ control) of at least 2 in all the last three time points.

1.67 % of the transcriptome (531 transcripts from 26,064 contigs) showed significant differential expression at the elevated temperatures with putative function for 177 transcripts, assigned by Blast sequence similarity searching against the NCBI non-redundant database, using an E-value cut off of 1e^−10^ (summarised in Additional file 1: Tables 1 and 2, for 25 °C and 21 °C, respectively). This annotation level of 23.6 % was similar to that obtained for other non-model organisms [25–27]. There was a considerable difference in the response of the scallops at the two temperatures, with only 95 transcripts differentially expressed in animals subjected to 21 °C, compared with 436 transcripts at 25 °C.

### Differentially expressed proteins

Using 2-DE, a mean proteome content of 956 spots was observed. Analyses revealed that 24 protein spots (2.5 % of the observable proteome) were significantly differentially expressed at 25 °C, at one or other of the time points (T4: 27 days or T7: 56 days), of which 12 were up-regulated and 12 were down-regulated (Table 1, 3). All of these 24 protein spots were subjected to trypsin digestion and Matrix-Assisted Laser Desorption Ionization Time-Of-Flight tandem mass spectrometry (MALDI TOF-TOF) analysis. This led to the identification of nine proteins using sequence similarity searching (Table 2). Of these nine putatively annotated proteins, five were identified from ESTs sequenced in the RNAseq experiments described in this study, and the other four proteins from the scallop hemocytes ESTs [28] used to build our database. This annotation level of 37 % was similar to proteomic studies in other non-model species [26, 29].

**Table 1:** Values of Log_2_ Fold Change for spots differentially expressed between animals maintained at 15 °C and animals maintained at 25 °C, after 27 days (T4) and 56 days (T5)

	Log_2_ FC (25 °C/15 °C)	
Spot	T4	T5
HiT-1	2.22	0.97
HiT-2	2.07	2.85
HiT-3	1.10	0.33
HiT-4	0.95	1.20
HiT-5	0.62	1.42
HiT-6	0.43	1.22
HiT-7	0.36	1.80
HiT-8	0.27	1.50
HiT-9	0.11	1.16
HiT-10	−0.50	1.11
HiT-11	−0.41	1.23
HiT-12	−1.14	1.42
LoT-1	−1.91	1.04
LoT-2	−2.31	0.98
LoT-3	−1.62	0.87
LoT-4	−2.14	0.80
LoT-5	−1.43	0.74
LoT-6	−1.77	0.38
LoT-7	−1.75	0.14
LoT-8	−1.50	−0.23
LoT-9	−1.37	−0.61
LoT-10	−0.14	−1.00
LoT-11	−0.30	−1.01
LoT-12	−0.33	−1.77

**Table 2:** List of *Pecten maximus* mantle tissue proteins identified by MS/MS whose abundance change between animals maintained at 15 °C and animals maintained at 25 °C (moderate *t*-test paired-comparison. fdr <0.1. absolute fold change >2)

Spot	Blast hit name	Acc. Nr	EST sequence name	Sequenced peptides	Peaks score
HiT-1	calumenin-B-like (CALUB)	XP_003704568	Contig22840	TEFMYFVHPEEGK	83.6
				DVTVCEYTDR	
HiT-2	calumenin-like isoform X3 (CALU)	XP_005110308	Contig_Reproseed325	DVLVLEYTDR	98.0
				DNAEEFEHATEQESK	
				CSWPNYVR	
HiT-9	No hit	-	Contig_Reproseed34795	TFVFPPADTQKPVITGIM(+15.99)R	88.0
				GESYIHVK	
				TFVFPPADTQKPVITGIMR	
				TFQM(+15.99)FDALQYIEQGNM(+15.99)IQGR	
LoT-2	Putative phosphoglycerate mutase (PGAM)	EKC26210	Contig_Reproseed34491	VLISAHGNSLR	94.3
				SYDVPPPAREDGDER	
				YAHKDASVVPR	
LoT-3	glyceraldehyde 3-phosphate dehydrogenase (GAPDH)	XP_002434347	scallop_rep_c46254	SSXFDANAGHAYNNNFVK	98.3
				VPVPDVSVVDHTC(+57.02)R	
				VVSQDFC(+57.02)GDSR	
LoT-6	gelsolin-like protein 2-like isoform X1 (GSN)	XP_005100380	Contig28884	AWDGAGQEPGTQFWR	85.7
				QSWQVGNR	
				NSGNSGDVYXPDGGR	
LoT-7	Kynurenine--oxoglutarate transaminase 3 (CCBL2)	EKC20610	Contig34767	NLGENFLR	97.3
				AVNSDNANWAQYAR	
				GQVPDDGSDDPYDYK	
				IASLPGM(+15.99)WDR	
LoT-9	peptidyl-prolyl cis-trans isomerase B precursor (PPIB)	NP_990792	Contig2622332	DFMVQGGDFSEGDGTGSK	98.4
				VSEGMDVVR	
				KLENTEVDIENR	
				YFADENFK	
LoT-11	Collagen alpha-6(VI) chain (COL6A6)	EKC27115	Contig35480	FNTNADQASVTEDVDDRR	99.0
				DDAEHVGIILTDGTTNPGR	
				REFNLDSYTKPAEVEQGLSNVR	
				NFQLELSFVK	
				ALTLLLDEGFTVR	
				VVSNFDIGSDNTR	

### Network analysis

**Fig. 4:**
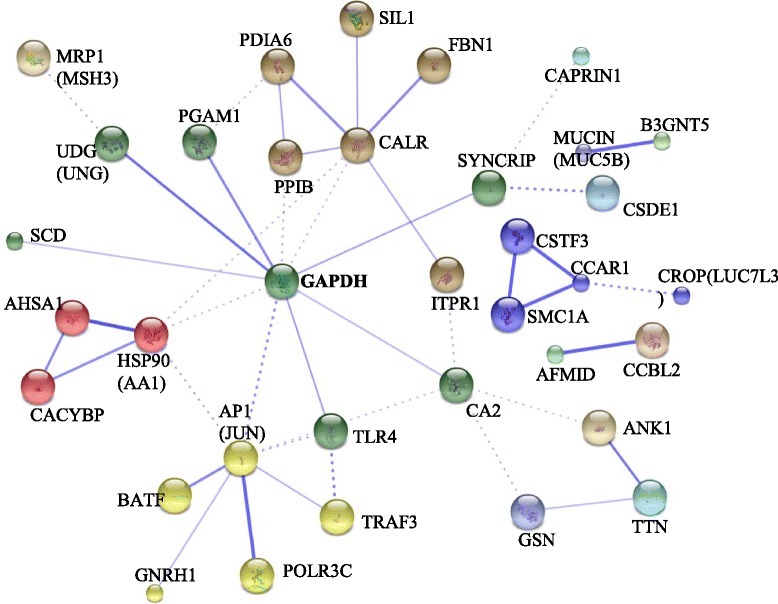
Network showing interactions of genes/proteins (*n*=134) found in this study. Network was constructed using the String 9.05 algorithm

All of the 177 transcripts putatively annotated using the initial Blast sequence similarity searching were used in further searches against the UniProt human database and the results (short names of genes) were entered into the STRING program. In all, 132 genes had homologs in human, and network analysis highlighted relationships between 35 of them (Fig. 4), among which only one, titin, was relied to the 21 °C condition, the other sequences were all from the 25 °C samplings. The main result showed a central node around glyceraldehyde-3-phosphate dehydrogenase (GAPDH: 10 interactants), which showed weaker links to nodes centered around the c-JUN pathway (8 interactants), calreticulin (CALR: 7 interactants), 90 kDa heat shock protein (HSP90: 5 interactants) and ankyrin (ANK1:3 interactants). Three other independent networks were also identified centering on the cell division cycle and apoptosis regulator (CCAR1), arylformamidase (AFMID) and mucin (MUCIN/MUC5B) (for full list of gene symbols and names see Additional file 1).

## Discussion

In this paper, we used both transcriptomic and proteomic analysis of mantle tissue to characterize the acclimation of *P. maximus* to 56-days exposure to to elevated thermal stress. The sea surface temperatures in the Bay of Brest vary annually between 10 and 18 °C (data from the Somlit-Brest, Coastal time-series station; 10 m depth), hence the choice of 15 °C as the control temperature, as this is the temperature in the Bay when the animals were brought into the aquaria. 21 °C was chosen as this represented the maximum temperature this species experiences in the region, with 25 °C as a more extreme, stressful temperature.

This range of temperatures is within those tested previously on *P. maximus* [15, 16, 30, 31]. Both the physiological data (condition index) and expression profiles in this experiment showed significant changes in animals kept at 25 °C compared with 21 °C. This is coherent with previous studies which show that *P. maximus* can rapidly acclimate (in timescales of days to weeks) to water temperatures below 23 °C [21, 30, 31]. The paucity of differential expression and stability of the condition index (Additional file 1: Table 2, Fig. 2) of the 21 °C animals indicates that these animals have successfully acclimated to this slightly elevated temperature [21]. This does not appear to be the case with the 25 °C animals where there is a reduction in condition index and this is the temperature which exhibits 82 % of the differentially expressed transcripts. Recently published experiments have shown that prolonged exposure, in excess of a week, to 25 °C can take *P. maximus* beyond their optimal thermal window with a switch towards anaerobic metabolism [15, 16]. These analyses also showed that at 25 °C *P. maximus* reduced respiration rates, indicating that metabolism was shutting down and also significant mortalities occurred when temperature was combined with hypoxia in a multistressor experiment [15, 16]. In the experiment described here there were no significant mortalities reported at 25 °C and thus network analysis of the differentially expressed transcripts and proteins at this temperature can provide insights into cellular responses to chronic heat stress. It was possible to partition these results into different nodes and functional categories, as described below.

### GAPDH as a pivotal protein in thermal acclimation

The network analysis placed GAPDH (glyceraldehyde-3-phosphate dehydrogenase) as a central node, linking several other pathways. GAPDH is a key enzyme in the glycolytic pathway, catalysing the conversion of glyceraldehyde-3-phosphate (G3P) to 1,3-biphosphoglycerate in the presence of NAD+ and inorganic phosphate. Therefore the presence of this transcript might not be unexpected due to the thermal dependency of biochemical reactions. Higher temperatures would cause an increase in the animals’ metabolic rate with glycolysis providing the additional energy needed, at least in the short term [32]. Indeed, evidence of up-regulation of glycolytic pathways has been identified in other molluscs thermal acclimation studies [4, 18]. Data from our study indicate that this scenario is unlikely, as there is a reduction in condition index (Fig. 2) and putative lipid metabolism transcripts are being up-regulated (see below). This indicates that carbohydrate sources have been exhausted and that stored energy reserves are being mobilised. Thus GAPDH may be playing a role in alternative biochemical pathways. Until recently, this enzyme was purely considered as a house-keeping gene. However recently, new extra-glycolytic roles for GAPDH in a diverse range of cellular processes have been discovered, such as binding nucleic acids, especially certain mRNAs and telomeric DNA [33]. It may also function as a transcription factor interacting with RNA pol II [34], and acts as a signaling protein, by regulating the activity of the mTOR signaling pathway through sequestering the GTPase Rheb [35]. Finally, several links between GAPDH and apoptosis have also been established. This is via the interaction of GADPH with the ubiquitin ligase Siah1, but it also plays a role in p53 and caspase-3 dependent apoptosis [33, 36].

GAPDH was only identified in the proteomic analysis and was not significantly up-regulated in the transcriptional profiling. However, it should be noted that GAPDH acts in cells as a homotetramer, and is known to be primarily regulated through post-translational modifications (e.g. phosphorylation; [37]). Whilst GAPDH was centrally positioned within the network, there were several links to a smaller node centered on the pleiotropic transcription factor AP1 (activating protein 1).

### A major role for signaling through the AP-1 pathway ?

Numerous genes encoding nuclear proteins, or involved in protein trafficking in the nucleus (Additional file 1: Tables 1 and 2), were found differentially expressed in this study, of which, AP1 (JUN) was highlighted by the network analysis. AP-1 is known to regulate a number of major physiological processes including cell fate. This may either be proliferation or apoptosis, with the cellular context critical for determining which pathway is activated [38, 39]. AP-1 is not a single protein, but a complex of different dimers from several groups of the Jun bZIP (basic region leucine zipper) domain proteins [40]. In this study, putative transcripts were identified for other potentially related CREB/ATF bZIP transcription factors. One of these transcripts showed high sequence similarity to BATF (Basic leucine zipper transcription factor, ATF-like), whilst the other matched a hypothetical protein containing a bZIP domain, thus suggesting that it belonged to the same class of transcriptional regulators.

Like all bZIP transcription factors, proteins within the AP-1 complex must dimerize before binding to their DNA target sites. However, each of the proteins leading to the AP-1 complex are differentially expressed and regulated, so every cell contains different mixtures of AP-1 dimers which can result in subtle differences in function [39, 40]. This is shown through the network analysis with links to ten other genes, known to be regulated by this transcriptional factor or interacting with it, including BATF, but also TLR4, GNRH, POLR3C, CA2, HSP90, TRAF3 and GAPDH. Whilst the network was built on interactions of human genes, in other species, AP1 has been found to regulate other genes such as gelsolin, collagen, phospholipases and DNA polymerases, some of which are differentially expressed in this study (additional file 1: Tables 1 and 2). Therefore it is entirely possible that some of these other transcripts are in fact members of the AP1 regulation pathway in *P. maximus*.

Additional links to AP1 were identified via several components of a multi subunit autoregulatory ribonucleoprotein complex, potentially involved in translationally coupled mRNA turnover [41]. This complex may associate with the mCRD domain of c-FOS (a component of AP-1), and included, PAIP1, HNRPD, CSDE1 and SYNCRIP. Putative homologues of the latter two genes, SYNCRIP and CSDE1, were found to be up-regulated at 25 °C in this study. Interestingly, SYNCRIP was also shown to interact with CAPRIN1 and with GAPDH in our network analysis. Of the links identified in the AP1 network, that with TRAF3 (tumor necrosis factor receptor (TNFR)-associated factor 3) is of particular interest. TRAF3 was one of the first identified TRAFs, initially isolated by virtue of its binding to the cytoplasmic domain of the TNFR-superfamily (TNFR-SF) member CD40 [42]. TRAF3 is a pivotal protein in cell signaling interacting with numerous receptors, such as TLR, RLR, CD40 etc. Interestingly, its action may require ubiquitination [43], and may lead to the activation of the JNK pathway and the negative regulation of NF-kappa B, which is involved in the immune response and cell atrophy [44]. AP-1 is major target of the JNK MAP kinase pathway and it is known to regulate a number of major physiological processes including cell fate. It is likely in this experiment that the putative AP-1 (JUN) transcript was involved in apoptosis, as the condition index of the animals was decreasing over time.

A similar link between members of the glycolytic pathway and apoptosis was identified in a recent transcriptomic study of chronic thermal challenge in the oyster *Crassostrea gigas* [4]. In that study glucose-6-phosphate translocase was identified in the transcriptional profiles along with several gene members involved in the mTOR signalling pathway and apoptosis [4]. Like the study described here, these profiles were associated with animals with reduced condition indexes and physiological condition, which were struggling to cope with exposure to elevated temperatures.

### Heat shock proteins

In our transcriptome data genes encoding both the 70 kDa (HSP70) and 90 kDa (HSP90) genes and a small HSP40 were up-regulated at 25 °C, along with several related genes, including an activator of HSP90 (AHSA1) and other molecular chaperones e.g. PPIB, PDIA, CALR (Additional file 1: Table 1). Proteins involved in proteins recycling (linked to ubiquitin dependent protein recycling) were also found. This indicates a cellular requirement for enhanced protein folding capacities and removal of mis-folded proteins, which are classic indicators of cell stress. To date, the only universal response to heat stress documented is the production of heat shock proteins (HSPs), often called the heat shock response (HSR) [45] and these genes are often monitored as a measure of environmental stress in molluscs [4, 13, 14, 17, 18, 22]. The majority of the experiments in scallops to date have used HSPs to test thermal tolerance, but these genes have largely been identified in acute exposures [13, 14, 21]. What is particularly interesting with this *P. maximus* experiment, is that putative HSPs and chaperone transcripts were still expressed after a chronic exposure and production of these proteins is known to be energetically costly [46].

However, the heat shock response is complex and dependent on the duration, type and intensity of the stress [47] and these proteins are not just involved in response to heat shock. HSPs have a wider role in relation to apoptosis and the inflammatory process [48]. Experimental induction of HSPs has been shown, in a variety of mammalian tissues, to inhibit apoptosis and considerably enhance cell survival [49, 50]. Thus, although many molluscs show expansions of heat shock gene family members [51, 52], these may not all be required for response to thermal stress. For example, the Pacific oyster may have up to 88 HSP70 genes in the genome, a study which exposed this species to elevated temperatures for 3 months, only identified a single HSP7012B and HSP60 gene in the expression data [4]. The poor numbers of HSPs identified as up- regulated in the oyster study and the continued expression of HSPs in our study might reflect the functional diversity of HSPs in molluscs.

### Long term-exposure to heat stress and apoptosis

The previous results, detailed above, on the activation of the AP-1 signalling pathway and up-regulation of HSPs, suggest a tension between cell renewal and apoptosis in the mantle tissue of 25 °C animals. The AP-1 complex can either promote cell proliferation or apoptosis and HSPs can have an anti-apoptotic function. In the transcriptome data a number of differentially expressed genes encoded apoptosis-related proteins. In particular, 4 transcripts were putatively annotated as inhibitors of apoptosis (3 were annotated as BIRC7, and 1 as PIAP) (Additional file 1: Table 1). The BIRC7 proteins repress apoptosis through the inhibition of CASP3, CASP7 and CASP9, and through their E3 ubiquitin-protein ligase activity. In addition, the major regulator of apoptosis (CCAR1) was also found up-regulated during heat stress. Network analysis showed interactions with CROP, which encodes a cisplatin resistance associated over-expressed protein (cisplatin induces cell death through induction of apoptosis [53], CSTF3 (Cleavage stimulation factor 77 kDa), and SMC1A (Structural maintenance of chromosomes protein 1A). A number of genes related to the ubiquitination and protein degradation were also up-regulated, which would be required if mis-folded proteins were accumulating. So the fact that after 56 days of being cultured at 25 °C HSPs and anti-apoptotic transcripts were significantly up-regulated in *P. maximus* indicates that the scallops were probably subject to chronic stress and having to prioritise the unfolded protein response either/and regulate apoptosis. The latter of which would certainly agree with the results showing a reduction in body mass, the decreased condition index and the mobilisation of energy reserves, as described below. This agrees with previous long-term studies in molluscs. In these, if the temperature increase was within the acclimation capacity of the animals, little response was seen in terms of gene expression profiles [21, 22], whereas lack of acclimation was demonstrated by a measurable cellular stress response [4, 17, 54].

### Heat stress and mobilisation of stored energy reserves

The transcription profiles suggest that the animals at 25 °C were struggling to consume and metabolise sufficient food to support life at the higher temperature. This agrees with the condition index (CI) data, which show a steady decrease in the CI of the 25 °C animals with time. When animals are subjected to increased temperatures, their metabolic rate also increases [55]. Our results suggest that they boost energy production to deal with this and also the increased energetic demand associated with mounting a robust stress response. Numerous studies have revealed the up-regulation of metabolism genes in hyperthermally stressed organisms such as the yeast *Saccharomyces cerevisiae* [56], the goby *Gillichthys mirabilis* [57], the mussels *Mytilus californianus* [58] and *M. trossulus* [18]. In these studies, most of genes related to energy metabolism, which were up-regulated, were related to glycolysis and the subsequent respiratory metabolism. This was not the case with the transcriptomic data described here. Two proteins related to the glycolysis (GAPDH and PGAM) were found to have their abundance decreased at 25 °C, while several genes associated with lipid metabolism were up-regulated, i.e. APMAP, PLA2G16 (2 contigs), AMACR, and ACOT4, suggesting a switch towards the mobilization of stored lipids. A similar observation was found in one of the few studies dealing with the adaptation to prolonged exposure to heat stress and low pH in a mollusc bivalves, the Pacific oyster [4].

### Remodelling of cell membranes and cytoskeleton

Changes to the basic cell structure were indicated by the differential expression of several transcripts, at both 21 °C and 25 °C (Additional file 1: Tables 1 and 2). It is well known that temperature affects the lipid constitution of cell membranes [59] as it is essential to maintain the membrane lipids in the liquid crystalline phase to maintain function. This is mainly achieved by altering the relative amounts of unsaturated fatty acids (UFAs) within the membranes, a process termed homeoviscous adaptation (HVA) [60]. In animals, the main enzyme involved in fatty acid desaturation is the enzyme ∆9-acyl-CoA desaturase. In our study, we showed that the gene encoding this enzyme (SCD) was strongly down-regulated in the mantle cells of scallops subjected to 25 °C. This would most probably result in a decreased level of unsaturation in membrane fatty acids, thus reducing the fluidity of membranes at the higher temperatures.

Cytoskeleton transcripts also showed differential expression at both 21 °C and 25 °C (Additional file 1: Tables 1 and 2). At the proteomic level several proteins involved in structure were also identified as decreased in abundance at 25 °C (GSN, COL6A6, Table 2). Beside from their structural role, cytoskeleton proteins have been shown to have a protective role in response to reactive oxygen species produced as a result of cell stress [61]. However a wider range of cytoskeletal proteins, such as tubulin, were implicated in studies investigating thermal stress in mussels, with a suggestion that this was due to the changing requirements for cell proliferation [18, 54, 62].

### DNA damage and repair

Our transcriptomic study revealed the differential regulation of numerous genes encoding proteins putatively involved in DNA repair and replication, both at 21 and 25 °C. In particular, we observed the down-regulation of not less than five different contigs annotated as Uracil DNA glycosylase (UDG). Numerous studies have indicated that environmental stress can affect genome stability in eukaryotes [63]. For example, in mammalian cells, hypoxia and starvation can suppress error-free DNA repair pathways (e.g. mismatch repair and homologous recombination) and cause an increase in mutagenesis [64–67] and Shor et al. [68] recently discovered that the yeast environmental stress response regulates mutagenesis. Base excision repair (BER) is one of the most important mechanism protecting cells from the deleterious effects of endogenous DNA damage induced by hydrolysis, reactive oxygen species or other intracellular metabolites that modify the DNA base structure. The pivotal enzymes involved in BER are DNA glycosylases, which remove different types of modified or damaged bases by cleavage of the N-glycosidic bond between the base and the 2-deoxyribose moieties of the nucleotide residues. Different DNA glycosylases, including UDG, remove different kinds of damage, and the specificity of the repair pathway is determined by the type of glycosylase involved. Uracil appears in DNA as a result of cytosine deamination, one of the most common DNA spontaneous modifications (that naturally occur in genomes at a rate of around 100 lesions per cell per day). Since this lesion is directly mutagenic, producing C → T transition, the production of UDG is essential for all living organisms. Therefore, the down-regulation of not less than five different contigs annotated as UDG in this study indicates that these cells may experience increased mutation rates, which could ultimately lead to increased cell death.

### Acute stress versus time limited thermal tolerance versus long term acclimation

Laboratory-based thermal tolerance trials can provide valuable data on species’ capacities to respond to changing environmental conditions, particularly their ability to acclimate [69]. If these studies include molecular analyses, then the cellular responses underlying sub-lethal exposures can identify which biochemical pathways are up regulated and therefore help predict energetic trade-offs and long–term survival [70]. To date, the majority of such multidisciplinary thermal trials on scallop species have involved acute exposures with the consequential up-regulation of heat shock proteins [13, 14]. The one longer term study of 14 days showed an immediate “classical” stress response, but by 14 days the animals appeared to have fully acclimated to the new temperature with gene expression levels returned to those of the controls [21]. This expression profile is typical of temperate species where the thermal stress applied is within the normal range experienced by that species and not extreme [22]. In this study we were primarily interested in long term effects and therefore did not examine molecular responses at acute timescales. The transcriptional profiles reflect this, with a lack of up-regulation of antioxidants which often accompany acute responses [5, 18, 21, 22]. The lack of response and stable condition index of the 21 °C animals mirrors the 14 days study [21] on the Zhikong scallop *Chlamys farreri* which demonstrated successful acclimation. It should be noted that adult acclimation does not necessarily guarantee stable reproductive capacity and successful recruitment of juveniles, longer term, generational studies would be required to test this. In contrast, the 25 °C animals showed many similarities in their expression profiles with a chronic 3 month exposure to elevated temperature in *C. gigas,* which in spite of a lack of animal mortality, showed a decrease in condition index and induction of apoptosis and mobilisation of stored energy reserves [4]. If both the experiment described here and the *C. gigas* study had been extended by several months, we would predict significant mortalities, as the animals are merely resisting the higher temperatures and are exhibiting time limited thermal tolerance.

## Conclusions

The aim of this study was to assess the molecular mechanisms underlying responses of the king scallop to prolonged heat stress. It was clear from both the condition indices and the molecular results that the scallops were able to support long-term culture at 21 °C, but 25 °C resulted in chronic cell stress, a reduction in animal mass and potential viability. Combining RNAseq based transcriptomics and proteomics approaches we identified differential expression in a number of different pathways. Whatever the temperature, the classical heat shock response appeared reduced. Rather, our main results suggested a remodelling of the cell structure as revealed by the differential expression of many genes involved in the cytoskeleton. Interestingly, several genes involved in DNA repair appeared down-regulated, suggesting that cells turned towards a mutator state in response to stress. We also showed, in animals maintained at 25 °C, modifications of cell membrane properties (through the modulation of fatty acids unsaturation) and an apparent mobilization of lipid energy reserves. Finally, our results also suggested a central role for the AP-1 signaling pathway and a potential tension with regard to apoptosis, and led us to propose a pivotal role for GAPDH, a common glycolytic enzyme which has recently been shown to play a critical role in cellular functioning. These are clearly key pathways to investigate in future studies into the thermal resilience of the king scallop *P. maximus.*

## Methods

### Biological material

Juveniles of *Pecten maximus* (1 year old; average length 34.0 mm ± 4.1 mm S.D.) used in this study came from the same cohort spawned at the Tinduff hatchery (Bay of Brest, France) in Spring 2010. They were then raised in small cages at Sainte-Anne du Portzic in the Bay of Brest for 1 year before being transferred to tanks at the Argenton Shellfish Laboratory (Brittany, France) of the French Research Institute for Exploitation of the Sea (IFREMER). Thus, all animals were similar in size, age and physiological condition.

### Experimental culture conditions

The scallops were randomly divided into three batches of 440 individuals placed into 250 L tanks. In each tank, the 440 individuals were sub-divided into two groups and placed in plastic cages without sediment. Each tank was supplied with 1 μm filtered, UV sterilized seawater at a controlled temperature of 15 °C (the temperature in the Bay of Brest at the time) with a flow rate of 80 L.h^−1^. A 12:12 photoperiod regime was used. Complete water changes were performed once a week. The animals were continuously fed with a cultured mix of microalgae (50 % *Isochrysis affinis galbana* (Tahitian strain *T.iso*) and 50 % *Chaetoceros calcitrans*) via a peristaltic pump delivering directly into the inflow water supply and mixed in the tanks by aquarium pumps. The supply of microalgae to each tank was regulated twice a day by counting the microalgae at the inlet and outlet of the tank with a Coulter counter to ensure that the outflow water contained 1.5 × 10^6^ μm^3^.mL^−1^ of microalgae [71]. This ensured that the scallops were fed *ad libitum* irrespective of the temperature and the number of individuals in the tank. The scallops were initially left at the ambient temperature of 15.1 ± 0.2 °C for 16 days to allow the animals to acclimatise to the rearing conditions and to identify any damaged or unhealthy individuals prior to experimentation. Then, one tank was maintained as a control at approximately 15 °C (15.1 ± 0.2 °C), while the temperature was increased at 1 °C/day to reach 21 °C (21.4 ± 0.2 °C) and 25 °C (25.2 ± 0.9 °C) in the two other tanks. During the experiment, the temperature was recorded every 15 min in the three tanks using button temperature data loggers (Signatrol SL52T Button Data Logger). Additional environmental parameters were also monitored: salinity, pH, O_2_ and ammonia. The condition index (CI) was estimated according to the Lucas and Beninger [72] method. For this, 15 to 30 animals were sampled every week. Soft tissues were dried at 75 °C during 48 h and shells were air-dried. Tissues and shells were weighted, and the condition index (CI) was calculated following the formula:

$$ CI = \left( Soft\  Tissues\  Dry\  Weight/ Shell\  Dry\  Weight\right)\times 100 $$

### Molecular analyses

All molecular analyses were carried out using the mantle tissue. The choice of mantle as the experimental tissue was influenced by several factors. A number of transcriptome studies have been published for this organ, which enhanced the annotation potential of transcripts [25, 27, 73]. Also, this organ has multiple functions including shell formation, secretion of the ligament and sensorial activities and hence is very responsive to external stimuli [74] and transcription profiles of the mantle can act as an effective proxy of early whole-animal response [4]. At the beginning of the experiment (T0), 10 (four for transcriptomic and six for proteomic) individuals were sampled and then, 10 individuals were sampled in each temperature treatment after 3 days (T1), 14 days (T2), 21 days (T3), 27 days (T4) and 56 days (T5). The scallops were quickly dissected and mantle tissue was flash frozen in liquid nitrogen and stored at −80 °C until further analysis.

### RNA extraction and sequencing

Total RNA was extracted from the mantle tissue of 4 individuals per condition at each time point using TRI Reagent® Solution (Life Technologies) according to manufacturer’s instructions (22 libraries in total). RNA from the four individuals was then pooled for each time point separately. RNA quality and concentration were determined using an Agilent 2100 RNA Nanochip (Agilent, Santa Clara, CA, USA) and by NanoDrop ND-1000 Spectrophotometer (260/280 nm, NanoDrop Technologies, Wilmington, DE, USA), respectively. Samples were then treated with a DNA-free Kit (Ambion, Austin, TX) to remove genomic DNA, and reverse transcription was carried out using 500 ng of total RNA, random hexamers, and MMLV reverse transcriptase (Promega) according to manufacturer’s protocols. The production of Illumina libraries for mRNA-seq and the transcriptome sequencing using Illumina HiSeq™ 2500 sequencing machine (HiSeq 100 pair-ends) was conducted by the Genome Analysis Centre (Norwich, UK), as described in [24].

### RNA-Seq data sets

The RNA libraries yielded a total of 806 million paired end reads. Raw reads were filtered and trimmed using the FASTX-toolkit (Version 0.0.13 from Assaf Gordon Hannon lab) and rRNA contamination removed using ribopicker [75] and cutadapt (Version 1.1; [76]), with a final quality check performed using fastQC (Version 0.10.0; http://www.bioinformatics.bbsrc.ac.uk/projects/fastqc/). The contigs were assembled using SOAPdenovo [77] and a kmer size of 89 was used to construct the initial de novo transcriptome assembly, resulting in 1,311,367 contigs, which were further assembled with CAP3 [78]. Redundancy was determined by self-Blasting and the use of CD-HIT (95 % similarity [79]). *De novo* assembly produced a total number of 26,064 contigs [24]. The transcripts were then processed through the Blast2GO pipeline to produce putative annotations and functional classifications based on Blastx results against the GenBank NR database release 190. To enable differential genes expression analyses, normalization was carried out by dividing counts by library size using TPM (Transcripts per million). Ratios were calculated for each time point with 25 °C *versus* 15 °C treatment and 21 °C versus 15 °C. Only the transcripts with a ratio higher than two successively at T3, T4 and T5 were kept for further analyses.

### Protein extraction and separation

Mantle tissues from six individuals from the 25 and 15 °C treatment at the two last time points (T4 and T5) were crushed with a mixer mill (MM400, RETSCH, Haan, Germany). 100 mg of this powder was homogenized in 100 mM Tris–HCl (pH 6.8) with 1 % of Protease inhibitor mix (GE Healthcare), centrifuged (4 °C, 50,000 g, 5 min) and supernatants removed to new tubes. Nucleic acids were then removed (nuclease mix, GE Healthcare, following manufacturer’s instructions). Samples were precipitated at 4 °C using TCA 20 % (1/1:v/v, overnight). After centrifugation (4 °C, 20,000 g, 30 min), pellets were washed with acetone 70 % and re-suspended in urea/thiourea buffer (2 M thiourea, 7 M urea, 4 % CHAPS, 1 % DTT) containing 1 % IPG (pH 4–7, GE Healthcare). Protein concentrations were determined using a modified Bradford assay [80], and all samples were adjusted to 400 μg of proteins in 250 μl. Prior to isoelectric focusing, IPG strips (pH 4–7, 13 cm, GE Healthcare) were passively rehydrated with 250 μl protein solution in wells for 14 h. Isoelectric focusing was conducted using the following protocol: 250 V for 15 min, 500 V for 2 h, gradient voltage increase to 1000 V for 1 h, gradient voltage increase to 8000 V for 2 h 30, 8000 V for 3 h, and reduced to 500 V (Ettan IPGphor3, GE Healthcare). To prepare for second dimension SDS-PAGE electrophoresis, strips were incubated in equilibration buffer (50 mM Tris–HCl pH 8.8, 6 M urea, 30 % glycerol, 2 % SDS and 0.002 % Bromophenol Blue) for two 15-min intervals, first with 1 g.l^−1^ dithiothreitol and then with 48 g.l^−1^ iodoacetamide. IPG strips were then placed on top of 12 % polyacrylamide gels, which were run in 10 °C thermo-regulated device (SE 600 Ruby, Amersham Biosciences) at 10 mA per gel for 1 h and then 30 mA per gel until complete migration. Gels were subsequently stained with Coomassie Blue (PhastGel, GE Healthcare) and destained in 30 % methanol and 7 % acetic acid. The resulting gels were scanned with a transparency scanner (Epson Perfection V700) in gray scale with 16-bit depth and a resolution of 400 dpi.

### Differential proteomics analysis

Images were aligned and spots were detected and quantified using the Progenesis SameSpots software (version 3.3, Nonlinear Dynamics) applying the automated algorithm. All detected spots were manually carefully checked and artifact spots were removed. Data were exported as volume raw values and statistical analyzes were conducted in R [81] using the packages prot2D [82] and Limma [83]. Data were normalized (quantile normalization) and the samples from the 25 °C treatment were paired-compared using moderated *t*-test to the samples from 15 °C treatment (control) for each sampling date (with 6 replicates per group). A global correction by false discovery rate (fdr; [84]) was used, in order to take into account multiple comparisons issues and paired-comparison correction. Spots with an FDR threshold lower than 0.1 and an absolute fold change superior to two were considered as differentially expressed.

### Mass spectrometry and protein identification

Proteins for which the abundance was significantly changed between 25 °C and 15 °C were excised from gels and prepared for mass spectrometry (MS) analysis, essentially as described in [85]. Briefly, gel pieces were first washed in 50 mM ammonium bicarbonate (BICAM), dehydrated in 100 % acetonitrile (ACN), vacuum-dried, and then rehydrated with BICAM containing 0.5 μg sequencing grade porcine trypsin (Promega). After overnight incubation at 37 °C, peptides were extracted from the gels by alternatively washing with 50 mM BICAM and ACN, and with 5 % formic acid and ACN. Between each step, the supernatants were pooled, and finally concentrated by evaporation using a centrifugal evaporator (SpeedVac). Samples were resuspended in trifluoroacetic acid (TFA; 0.1 % in water), and mixed with the α-cyano-4-hydroxycinnamic acid (HCCA, 10 mg.ml^−1^ of a ACN/TFA/water (60/4/36:v/v/v) solution), and spotted on a polished steel target using the dried droplet method. Peptides were then analyzed by Matrix-Assisted Laser Desorption Ionization Time-Of-Flight tandem mass spectrometry (MALDI TOF-TOF) in positive ion reflector mode, using an Autoflex III (Bruker Daltonics) mass spectrometer. The FlexControl software (v3.0, Bruker Daltonics) was set up to acquire successively PMF spectra and MS/MS from the dominant peaks. Mass spectra were analyzed with FlexAnalysis (v 3.0; Bruker Daltonics) by applying the following conditions: TopHat algorithm for baseline subtraction, Savitzky-Golay analysis for smoothing (0.2 m/z; number of cycles: 1) and SNAP algorithm for peaks detection (signal-to-noise ratio: 6 for MS and 1.5 for MS/MS). The charge state of the peptides was assumed to be +1. Fragments of porcine trypsin were used for internal mass calibration. Proteins were subsequently identified with PEAKS (v.5.2, Bioinformatics Solutions) using MS/MS-based identification and *de novo* sequencing. The search parameters against a custom-made database were set as follows: carbamidomethylation of cysteine was set as a fixed modification, oxidation of methionine and phosphorylation of serine, threonine and tyrosine were set as variable modifications, one missing cleavage during trypsin digestion was allowed and the tolerance for precursor-ion mass tolerance was set to 1 Da. The database was constructed by combining *P. maximus* sequences from two sources: sequences acquired during this study [24] and sequences from a recent transcriptomic analysis of *P. maximus* hemocytes cells [28]. Overall, the database included a total of 252,888 *P. maximus* expressed sequence tags (ESTs). Protein identification was considered as unambiguous when a minimum of two peptides matched with a minimum score of 20. False discovery rates were also estimated using a reverse database as decoy. EST database sequences were annotated by sequence similarity searches against a non-redundant database using the Blast algorithm from NCBI (http://www.ncbi.nlm.nih.gov/BLAST).

### Network analysis

To better understand the relationships between the proteins and genes identified in this study,

network analyses using the String 9.05 algorithm [86] (http://string-db.org) were performed. STRING is a database of known and predicted protein interactions, which includes both direct (physical) and indirect (functional) associations. STRING quantitatively integrates interaction data and calculate a score for each protein interaction based on the genomic context, high-throughput experiments, co-expression and literature. This kind of network analysis is strongly dependent on the quality of the used database, and is thus much more efficient using databases from the most studied model organisms. Hence, the putative human homologues of the genes and proteins up- or down- regulated in this study were identified and used as the input data for the STRING program.

### Availability of supporting data

The sequence data related to this work has been deposited in the GenBank SRA, accession number: SRP040427. The contigs, and annotation are available from http://ramadda.nerc-bas.ac.uk/repository/entry/show/Polar+Data+Centre/NERC-BAS+Datasets/Genomics/.

## Additional file

Additional file 1:
**Table S1.** List of genes found deregulated at 25 °C. E-values, gene names and accession numbers are those given by Blast2GO for the best hit. Short names are, when possible, those of the best human homolog of the best hit (these names were used for String 9.05 analyses). Induction factors are given as Log2. **Table S2.** List of genes found deregulated at 21 °C. E-values, gene names and accession numbers are those given by Blast2GO for the best hit. Short names are, when possible, those of the best human homolog of the best hit (these names were used for String 9.05 analyses). Induction factors are given as Log2. (DOCX 251 kb)

## Abbreviations

MT: Metallothionein
HSP: Heat shock protein
2-DE: Two-dimensional protein electrophoresis
Q-PCR: Quantitative PCR
NGS: Next-Generation Sequencing
MALDI TOF-TOF: Matrix-assisted laser desorption ionization time-of-flight tandem mass spectrometry
EST: Expressed sequence tag
GAPDH: Glyceraldehyde 3 phosphate dehydrogenase
CALR: Calreticulin
ANK1: Ankyrin
CCAR1: Cell division cycle and apoptosis regulator
AFMID: Arylformamidase
G3P: Glyceraldehyde-3-phosphate
bZIP: Basic region leucine zipper
BATF: Basic leucine zipper transcription factor, ATF like
CREB: cAMP response element-binding
TLR4: Toll-like receptor 4
GNRH: Gonadotropin-releasing hormone
CA2: Carbonic anhydrase 2
TRAF: Tumor necrosis factor receptor associated protein
PAIP1: Polyadenylate binding protein-interacting protein 1
HNRPD: Heterogeneous nuclear ribonucleoprotein D
CSDE1: Cold shock domain containing E1
SYNCRIP: Heterogenous nuclear ribonucleoprotein
JNK: c-JUN N-terminal kinase
CASP: Caspase
UDG: Uracil DNA glycosylase
CAN: Acetonitrile
SDS: Sodium dodecyl sulfate
DTT: Dithiothreitol
BICAM: Ammonium bicarbonate
FDR: False discovery rate
TFA: Trifluoroacetic acid
HCCA: α-cyano-4-hydroxycinnamic acid
PMF: Peptide mass fingerprinting
MS/MS: Tandem mass spectrometry
