# Supplementary material for: Deciphering the molecular adaptation of the king scallop (Pecten maximus) to heat stress using transcriptomics and proteomics
Source: BMC Genomics. 2015 Nov 23;16:988. doi: 10.1186/s12864-015-2132-x (PMC4657243; doi:10.1186/s12864-015-2132-x)
Supplement: Additional file 1: — Table S1. List of genes found deregulated at 25 °C. E-values, gene names and accession numbers are those given by Blast2GO for the best hit. Short names are, when possible, those of the best human homolog of the best hit (these names were used for String 9.05 analyses). Induction factors are given as Log2. Table S2. List of genes found deregulated at 21 °C. E-values, gene names and accession numbers are those given by Blast2GO for the best hit. Short names are, when possible, those of the best human homolog of the best hit (these names were used for String 9.05 analyses). Induction factors are given as Log2. (DOCX 251 kb) [file 12864_2015_2132_MOESM1_ESM.docx]

Supplemental data

**Table S1: List of genes found deregulated at 25°C. E-values, gene names and accession numbers are those given by Blast2GO for the best hit. Short names are, when possible, those of the best human homolog of the best hit (these names were used for String 9.05 analyses). Induction factors are given as Log2.**

| **Functional Classification** | **Gene Name** | **Contig ID** | **E-value** | **Id (%)** | **Acc** | **Short Name** | **3 d** | **14 d** | **21 d** | **27 d** | **56 d** | **25°C** | |
| --- | --- | --- | --- | --- | --- | --- | --- | --- | --- | --- | --- | --- | --- |
| Cytoskeleton/ Cell Binding | Protocadherin-11 X-linked | Contig14009 | 0 | 67 | EKC36072 | PCD11X | -0.95 | 0.48 | 1.48 | 1.38 | 1.38 | up |  |
|  | Putative tubulin polyglutamylase TTLL2 | Contig24823 | 8.96E-58 | 78 | EKC20821 | TTLL2 | -2.23 | 0.50 | 1.11 | 1.69 | 1.73 | up |  |
|  | hypothetical protein CGI_10013101 | Contig31880 | 1.15E-80 | 72 | EKC18364 | PH-like | -1.91 | 0.69 | 1.67 | 1.21 | 1.47 | up |  |
|  | Ankyrin-1 | Contig4777 | 9.70E-28 | 56 | EKC31490 | ANK1 | -1.35 | -0.30 | 1.09 | 1.46 | 1.65 | up |  |
|  | intraflagellar transport protein 20 homolog | Kmer2588320 | 4.89E-45 | 84 | XP_798031 | IFT20 | -0.24 | 0.55 | 1.68 | 1.68 | 1.05 | up |  |
|  | Neurofilament heavy polypeptide. partial | Contig31123 | 1.19E-33 | 50 | ELR55547 | NEFH | -3.73 | 1.75 | -1.08 | -1.63 | -2.56 | down |  |
|  | cornifelin-like protein | Kmer2590676 | 1.46E-20 | 56 | XP_002730715 |  | -1.77 | -0.67 | -2.56 | -1.33 | -3.26 | down |  |
|  | Fibrillin-1 | Kmer2618224 | 1.66E-24 | 50 | ELK31446 | FBN1 | -1.70 | 0.31 | -1.27 | -2.43 | -1.51 | down |  |
|  | hypothetical protein CAPTEDRAFT_111329. partial | Contig34524 | 1.38E-33 | 57 | ELT87078 | FBN3 | 3.35 | 1.37 | 1.16 | 1.86 | 2.65 | up |  |
|  | neogenin-like | Contig35282 | 7.97E-57 | 57 | ELU03642 |  | 4.14 | -6.90 | -3.15 | -4.30 | -4.78 | down |  |
|  | Tenascin-X | Contig24522 | 3.13E-42 | 54 | EKC28247 | TNX | -0.84 | 2.61 | 3.82 | 2.80 | 3.57 | up |  |
|  | Keratin-associated protein 10-8 | Contig32243 | 2.67E-07 | 45 | XP_002719196 | KRTAP10-8 | -0.54 | 1.26 | 2.01 | 1.31 | 1.13 | up |  |
|  | Collagen alpha-5(VI) chain | Contig34595 | 2.73E-83 | 50 | EKC40283 | COL6A5 | -3.04 | -1.00 | -1.25 | -2.10 | -2.08 | down |  |
|  | hypothetical protein (mucin like) | Contig5092 | 6.40E-25 | 50 | XP_001283171 |  | -4.94 | 0.15 | -1.28 | -4.51 | -1.96 | down |  |
|  | Tenascin-X | Contig26966 | 2.00E-18 | 42 | EKC34579.1 | TNX | -2.16 | 0.47 | 2.42 | 3.31 | 6.13 | up |  |
|  | predicted protein (protocadherin 16) | Kmer2596774 | 1.24E-13 | 50 | XP_001632533 | DCHS1 | 0.18 | 0.70 | 1.59 | 1.17 | 2.39 | up |  |
|  | hypothetical protein CAPTEDRAFT_224751 (Lachesin) | Contig22445 | 3.22E-36 | 52 | ELT92279 | LAC | -1.88 | 1.90 | 1.29 | 1.65 | 2.12 | up |  |
|  | hypothetical protein CGI_10012792 | Contig29817 | 1.29E-142 | 80 | EKC28948 |  | -0.79 | 1.73 | 1.11 | 1.08 | 1.89 | up |  |
|  | Leucine-rich repeat and fibronectin type III domain-containing protein 1-like protein | Contig34753 | 1.17E-08 | 52 | EKC34931 | LRFN2 | -0.64 | 1.56 | 1.84 | 1.79 | 1.06 | up |  |
|  | Neuroglian | Contig30563 | 9.94E-115 | 69 | EKC24451 | NRG | -1.10 | 1.15 | 1.10 | 1.70 | 1.18 | up |  |
|  |  |  |  |  |  |  |  |  |  |  |  |  |  |
|  | AP-1 protein | Contig14186 | 2.57E-61 | 66 | ADZ48236 | AP1 | -0.93 | 0.91 | 1.20 | 1.82 | 1.52 | up |  |
|  | CREB/ATF bZIP transcription factor | Contig6686 | 4.32E-11 | 68 | EKC41122 | CREBZF | -2.22 | 1.72 | 1.99 | 1.56 | 1.77 | up |  |
|  | CREB/ATF bZIP transcription factor | Contig13363 | 2.10E-19 | 53 | EKC41122 | CREBZF | -1.04 | 1.81 | 1.97 | 2.51 | 1.98 | up |  |
|  | Basic leucine zipper transcriptional factor ATF-like 3 | Contig24201 | 1.82E-05 | 59 | EKC39151 | BATF | -0.73 | 3.34 | 1.60 | 4.42 | 2.65 | up |  |
|  | Cold shock domain-containing protein E1 | Contig21934 | 0 | 81 | EKC28502 | CSDE1 | -1.31 | 1.02 | 1.56 | 1.51 | 1.56 | up |  |
|  | WD repeat-containing protein 55. partial | Contig23964 | 6.40E-158 | 85 | EKC18506 | WDR55 | -1.14 | 0.87 | 1.49 | 1.63 | 1.52 | up |  |
|  | Conserved domain : NTF2 | Contig2406 | 1.70E-06 | 63 | ELT94896 | NTF2 | -1.72 | -0.04 | 1.16 | 1.54 | 1.41 | up |  |
|  | hypothetical protein CGI_10024367 | Contig24111 | 1.62E-35 | 54 | XP_002741750 |  | -3.10 | 1.11 | 2.54 | 2.16 | 2.68 | up |  |
|  | Pre-mRNA-splicing factor SYF1 | Contig25944 | 0 | 92 | EKC32877 | SYF1 | -1.70 | 0.70 | 1.56 | 1.47 | 1.55 | up |  |
|  | hypothetical protein CGI_10010503 | Contig27155 | 9.91E-102 | 64 | EKC23497 |  | -1.74 | 0.15 | 1.04 | 1.04 | 1.30 | up |  |
|  | Helix-loop-helix protein delilah | Contig34688 | 3.63E-25 | 87 | EKC41081 | DEI | -0.33 | 1.13 | 1.64 | 1.55 | 1.23 | up |  |
|  | Transposable element Tcb2 transposase | Contig34873 | 9.99E-15 | 69 | EKC40022 | TCB2 | -1.02 | 0.80 | 1.61 | 1.56 | 1.30 | up |  |
|  | Cisplatin resistance-associated overexpressed protein | Contig35733 | 4.22E-118 | 86 | EKC39141 | CROP | -1.28 | 0.34 | 1.22 | 1.49 | 1.16 | up |  |
|  | Nucleolar GTP-binding protein 1 | Contig4773 | 1.43E-60 | 80 | EKC19642 | GTPBP1 | -0.68 | 1.06 | 2.41 | 2.50 | 1.84 | up |  |
|  | Sin3 histone deacetylase corepressor complex component SDS3 | Kmer2605584 | 1.37E-82 | 84 | EKC32335 | SDS3 | -0.39 | 0.61 | 1.65 | 1.62 | 1.20 | up |  |
|  | HIV Tat-specific factor 1 homolog isoform 2 | Kmer2605766 | 2.45E-73 | 75 | XP_003727819 | HTATSF1 | -2.35 | 0.86 | 2.49 | 2.70 | 2.12 | up |  |
|  | Oxidoreductase HTATIP2 | Contig17480 | 1.00E-73 | 49 | EKC37161 | HTATIP2 | -0.08 | 1.19 | 1.64 | 1.99 | 1.51 | up |  |
|  | Hairy/enhancer-of-split related with YRPW motif protein | Contig20067 | 6.60E-18 | 52 | EKC31110 | HEY1 | 1.55 | 2.55 | 1.55 | 1.37 | 1.66 | up |  |
|  | Cleavage stimulation factor 77 kDa subunit | Contig17300 | 2.89E-139 | 85 | EKC20054 | CSTF3 | 1.04 | 0.09 | 2.21 | 1.81 | 1.14 | up |  |
|  | Cleavage stimulation factor 77 kDa subunit | Kmer2612942 | 1.74E-129 | 87 | EKC20054 | CSTF3 | 0.78 | -0.74 | 1.70 | 1.31 | 1.03 | up |  |
|  | Histone H1-delta | Kmer2595300 | 1.33E-22 | 84 | EKC17653 | H1 | -1.17 | 0.69 | 2.25 | 1.48 | 1.43 | up |  |
|  | Dermal papilla-derived protein 6-like protein | Kmer2612148 | 2.19E-62 | 63 | EKC36760 | DERP6 | -2.56 | -0.79 | 3.17 | 1.26 | 1.52 | up |  |
|  | Zinc finger protein 26 | Contig27896 | 3.41E-48 | 71 | EKC19913 | ZNF26 | -2.05 | 0.82 | 1.02 | 1.57 | 1.94 | up |  |
|  | eukaryotic translation initiation factor 5A-1 | Contig31784 | 1.75E-64 | 81 | NP_998350 | EIF5A | -2.62 | 0.59 | 2.55 | 3.36 | 4.53 | up |  |
|  |  |  |  |  |  |  |  |  |  |  |  |  |  |
|  | NTF2-related export protein 2 | Contig24619 | 1.18E-46 | 83 | EKC36366 | NXT2 | -1.77 | 1.55 | 1.06 | 1.14 | 1.43 | up |  |
|  | hypothetical protein CGI_10001573 (b_ZIP superfamily protein) | Contig26918 | 2.05E-38 | 56 | EKC27007 |  | -2.62 | 1.16 | 1.10 | 1.08 | 1.76 | up |  |
|  | HSPC148 | Contig27021 | 2.32E-66 | 80 | AAF29112 | HSPC148 | -1.42 | 1.20 | 1.00 | 1.09 | 1.00 | up |  |
|  | Transcription elongation regulator 1 | Contig29941 | 3.23E-88 | 89 | EKC23634 | TCERG1 | -2.94 | 1.58 | 1.06 | 1.36 | 1.04 | up |  |
|  | Caprin-1 | Contig30972 | 7.52E-54 | 76 | EKC23296 | CAPR1 | -1.24 | 1.35 | 1.66 | 1.11 | 1.64 | up |  |
|  | Heterogeneous nuclear ribonucleoprotein Q | Contig12409 | 4.01E-39 | 92 | EKC37135 | SYNCRIP | 1.04 | 0.93 | 1.83 | 2.80 | 1.03 | up |  |
|  | RNA-binding protein (2nd hit = hn-RNP Q) | Contig12883 | 2.55E-65 | 92 | ABP04054 | SYNCRIP | 0.88 | 1.60 | 2.58 | 2.87 | 1.54 | up |  |
|  | high mobility group protein D | Contig34400 | 5.94E-17 | 76 | BAM17877 | HMGD | -1.04 | 0.30 | 1.16 | 2.34 | 1.17 | up |  |
|  | Zinc finger CCCH domain-containing protein 3 | Contig35254 | 2.66E-58 | 59 | EKC35995 | ZC3H3 | -0.68 | 1.34 | 1.48 | 2.27 | 1.82 | up |  |
|  | hypothetical protein CGI_10006979 (N151 protein) | Contig11211 | 9.29E-34 | 93 | BAI49993 | N151 | -1.46 | 0.93 | 1.41 | 1.37 | 1.48 | up |  |
|  | N151 | Contig35661 | 5.35E-09 | 69 | BAI49993 | N151 | 0.23 | 1.19 | 1.16 | 3.26 | 2.18 | up |  |
|  | U11/U12 small nuclear ribonucleoprotein 25 kDa protein | Kmer2606506 | 2.45E-53 | 83 | EKC41736 | SNRNP25 | -1.89 | 0.93 | 1.08 | 1.75 | 1.08 | up |  |
|  | hypothetical protein CAPTEDRAFT_222987 | Kmer2612936 | 5.84E-78 | 71 | ELT89986 |  | -3.01 | 0.50 | 1.27 | 2.26 | 1.33 | up |  |
|  | Putative ATP-dependent RNA helicase DDX27 | Kmer2614026 | 5.16E-96 | 78 | EKC21239 | DDX27 | -1.55 | -0.60 | 1.41 | 3.06 | 1.48 | up |  |
|  |  |  |  |  |  |  |  |  |  |  |  |  |  |
|  | Poly [ADP-ribose] polymerase 14 | Contig30195 | 2.60E-29 | 60 | EKC32942 | PARP14 | -2.88 | 0.68 | 1.80 | 1.76 | 1.80 | up |  |
| Replication / DNA Repair | MRG-binding protein | Contig31296 | 2.49E-38 | 68 | EKC26944 | MRGBP | -2.15 | 0.69 | 1.50 | 1.73 | 1.30 | up |  |
|  | DNA-directed DNA polymerase epsilon 3 | Contig33716 | 7.65E-52 | 92 | NP_001140051 | POLE3 | -1.27 | 0.16 | 1.12 | 1.22 | 1.15 | up |  |
|  | pol-like protein | Contig14232 | 3.16E-67 | 57 | BAC82626 |  | -4.77 | 1.38 | -1.88 | -1.75 | -3.82 | down |  |
|  | endonuclease-reverse transcriptase | Contig29988 | 7.32E-29 | 63 | EHJ67413 | RTase | -1.72 | 0.31 | -4.81 | -2.86 | -3.13 | down |  |
|  | uracil-DNA glycosylase | Contig33560 | 6.09E-14 | 66 | YP_001219471 | UDG | 0.04 | 0.59 | -1.58 | -4.54 | -2.96 | down |  |
|  | predicted protein | Contig29412 | 1.01E-102 | 74 | XP_001632570 |  | 1.04 | 0.78 | 2.21 | 2.10 | 1.31 | up |  |
|  | uncharacterized protein LOC764672 | Contig31538 | 4.83E-29 | 57 | XP_003724962 |  | -2.62 | 1.00 | 4.51 | 2.24 | 1.77 | up |  |
|  | unnamed protein product | Kmer2589196 | 6.27E-92 | 87 | BAC34934 |  | -1.81 | -2.10 | 1.73 | 1.08 | 1.02 | up |  |
|  | CWF19-like protein 1 | Kmer2601862 | 9.39E-75 | 73 | EKC30101 | CWF19L1 | -1.94 | 1.49 | 2.32 | 1.05 | 1.33 | up |  |
|  | Uracil-DNA glycosylase | Contig21363 | 2.08E-60 | 70 | EKC38003 | UDG | -4.04 | 0.09 | -1.93 | -1.11 | -1.70 | down |  |
|  | hypothetical protein | Contig34404 | 8.57E-47 | 44 | AEO32622 |  | -2.05 | -1.95 | 1.06 | 1.06 | 3.70 | up |  |
|  | pol-like protein | Kmer2621110 | 4.10E-53 | 47 | BAC82624 |  | 0.23 | 1.57 | 2.23 | 2.08 | 3.75 | up |  |
|  | Exonuclease 3'-5' domain-containing protein 2 | Contig21482 | 6.73E-114 | 60 | EKC41618 | EXD2 | -1.12 | 1.48 | 1.29 | 1.67 | 1.31 | up |  |
|  | Putative RNA-directed DNA polymerase from transposon BS | Contig22409 | 2.49E-18 | 56 | EKC31648 | RTase | -0.99 | 1.48 | 1.27 | 1.54 | 1.53 | up |  |
|  | uncharacterized protein LOC101238120 | Contig29133 | 2.72E-55 | 66 | XP_004208670 |  | -4.60 | 2.70 | 2.09 | 2.47 | 3.20 | up |  |
|  | polymerase (RNA) III (DNA directed) polypeptide C (62kD)-like | Contig28898 | 7.25E-88 | 72 | XP_002740482 | POLR3C | -0.98 | 0.04 | 1.24 | 1.78 | 1.11 | up |  |
|  | Structural maintenance of chromosomes protein 1A | Kmer2615746 | 1.30E-158 | 88 | EKC29388 | SMC1A | -1.47 | 0.46 | 1.29 | 2.16 | 1.04 | up |  |
|  |  |  |  |  |  |  |  |  |  |  |  |  |  |
|  | hypothetical protein BRAFLDRAFT_128896 | Contig20242 | 1.03E-43 | 49 | XP_002611771 |  | -0.40 | 1.05 | 2.09 | 2.05 | 1.47 | up |  |
| Protein modification/ maturation | Peptide-N(4)-(N-acetyl-beta-glucosaminyl)asparagine amidase | Contig28726 | 4.65E-97 | 80 | EKC20424 | NGLY1 | -1.13 | 1.07 | 1.34 | 1.51 | 1.87 | up |  |
|  | ariadne-1-like protein | Contig28961 | 1.30E-24 | 49 | EKC29163 | ARIH1 | -3.56 | 0.82 | 2.24 | 1.59 | 2.51 | up |  |
|  | hypothetical protein CGI_10019403 | Contig29154 | 1.40E-38 | 73 | EKC37854 |  | -1.70 | 0.31 | 2.04 | 2.19 | 2.39 | up |  |
|  | hypothetical protein CGI_10024659 | Contig32670 | 4.16E-88 | 82 | EKC42154 |  | -1.89 | 0.40 | 1.16 | 1.20 | 1.17 | up |  |
|  | Carbohydrate sulfotransferase 15 | Contig3509 | 5.48E-23 | 61 | EKC27439 | CHST15 | -0.93 | 0.00 | 1.35 | 1.34 | 1.23 | up |  |
|  | dolichyl-phosphate beta-glucosyltransferase | Kmer2588360 | 4.19E-11 | 89 | NP_001080634 | ALG5 | 0.23 | -0.07 | 1.24 | 1.66 | 1.55 | up |  |
|  | E3 ubiquitin-protein ligase MIB2 | Kmer2591220 | 3.16E-36 | 70 | EKC39101 | MIB2 | 0.06 | 0.38 | 1.19 | 1.69 | 1.74 | up |  |
|  | UDP-GlcNAc:betaGal beta-1.3-N-acetylglucosaminyltransferase 5 | Kmer2611298 | 2.74E-10 | 60 | CAX69340 | B3GNT5 | -1.94 | -1.59 | -2.02 | -2.21 | -3.06 | down |  |
|  | Carbohydrate sulfotransferase 15 | Contig31979 | 8.13E-41 | 67 | EKC36000 | CHST15 | -0.15 | -0.13 | 2.83 | 1.06 | 2.04 | up |  |
|  | hypothetical protein CGI_10007323 | Kmer2588384 | 6.80E-56 | 77 | EKC23848 |  | -1.77 | 1.03 | 1.38 | 1.99 | 1.36 | up |  |
|  | SIL1 protein-like | Kmer2589504 | 6.44E-31 | 73 | XP_002737127 | SIL1 | -4.26 | 1.47 | 1.35 | 2.25 | 1.49 | up |  |
|  | Serine/threonine-protein kinase SBK1 | Kmer2616510 | 1.67E-176 | 86 | EKC36229 | SBK1 | -0.20 | 0.70 | 1.32 | 2.15 | 1.74 | up |  |
|  |  |  |  |  |  |  |  |  |  |  |  |  |  |
|  | FAM50-like protein | Contig27547 | 9.41E-112 | 89 | EKC29307 | FAM50 | -0.51 | 0.78 | 1.76 | 2.24 | 1.86 | up |  |
| Signaling | Calcyclin-binding protein | Contig30000 | 7.08E-39 | 65 | EKC28333 | CACYBP | -2.23 | 1.39 | 1.64 | 2.18 | 1.70 | up |  |
|  | Ras-related protein Rab-9A | Kmer2616396 | 2.79E-68 | 90 | EKC42702 | RAB9A | -0.42 | 1.06 | 1.44 | 1.36 | 1.47 | up |  |
|  | Ectonucleotide pyrophosphatase/phosphodiesterase family member 5 | Contig27767 | 5.22E-71 | 62 | EKC40323 | ENPP5 | -1.87 | 0.95 | -1.09 | -2.01 | -1.69 | down |  |
|  | L-rhamnose-binding lectin CSL3 | Kmer2588308 | 5.04E-11 | 80 | EKC38532 | CSL3 | -1.43 | -2.96 | -2.15 | -1.71 | -2.30 | down |  |
|  | tumor necrosis factor receptor-associated factor 3 | Contig22790 | 4.97E-17 | 75 | AFL03408 | TRAF3 | -0.90 | 2.64 | 1.93 | 1.64 | 1.99 | up |  |
|  | hypothetical protein CGI_10008481 | Contig28040 | 2.45E-148 | 72 | EKC19449 |  | -0.83 | 2.54 | 1.86 | 1.24 | 2.36 | up |  |
|  | hypothetical protein BRAFLDRAFT_123623 | Contig28118 | 6.62E-16 | 63 | XP_002590580 |  | -1.65 | -0.78 | -1.24 | -1.56 | -1.21 | down |  |
|  | Neurocalcin-like protein | Contig24768 | 2.46E-49 | 66 | EKC27427 | NCALD | -1.37 | 0.27 | 1.05 | 1.11 | 2.39 | up |  |
|  | similar to tumor necrosis factor receptor-associated factor 3 | Contig30315 | 1.43E-53 | 72 | EKC42493 | TRAF3 | -0.86 | 1.57 | 1.40 | 2.07 | 3.50 | up |  |
|  | canopy-like protein 2 | Contig32516 | 1.15E-56 | 70 | EKC42608 | CNPY2 | -2.94 | 1.07 | 1.49 | 1.25 | 2.38 | up |  |
|  | calumenin-B-like | Contig22840 | 2.00E-47 | 45% | XP_005816427 | CALUB | -1.66 | 3.87 | 3.59 | 3.10 | 3.40 | up |  |
|  | Teneurin-3 | Contig33008 | 1.30E-11 | 47 | EKC39942 | TENM3 | -0.62 | 1.67 | 1.85 | 1.68 | 1.23 | up |  |
|  | UPF0556 protein C19orf10 (interleukine 25)-like protein | Contig31721 | 1.98E-13 | 51 | EKC42343 | IL25 | -2.02 | 1.14 | 1.44 | 1.09 | 1.77 | up |  |
|  | tumor necrosis factor receptor-associated factor 3 | Contig20819 | 9.21E-128 | 61 | AFL03408 | TRAF3 | -1.30 | 2.19 | 1.34 | 2.90 | 2.15 | up |  |
|  | toll-like receptor D | Contig33472 | 3.11E-37 | 51 | AFU48616 | TLR4 | -0.43 | 1.28 | 2.13 | 2.60 | 1.86 | up |  |
|  | 28 kDa heat- and acid-stable phosphoprotein | Contig35358 | 2.17E-27 | 70 | EKC25746 | PDAP1 | -0.83 | 0.90 | 1.05 | 1.86 | 1.59 | up |  |
|  | calmodulin-like protein | Contig4442 | 6.28E-66 | 82 | XP_003766688 | CALM5 | 0.16 | 0.63 | 1.10 | 2.22 | 1.34 | up |  |
|  |  |  |  |  |  |  |  |  |  |  |  |  |  |
|  |  |  |  |  |  |  |  |  |  |  |  |  |  |
|  | Cubilin | Contig14685 | 4.47E-22 | 42 | EKC18187 | CUBN | -2.03 | 0.33 | 1.12 | 1.09 | 1.55 | up |  |
| Metabolism/ Energy related | Kynurenine formamidase | Contig26371 | 3.00E-45 | 62 | EKC35917 | KFASE | -1.50 | 2.16 | -1.22 | -1.47 | -1.77 | down |  |
|  | DDI1-like protein 2 | Contig25538 | 6.47E-31 | 74 | EKC18588 | DDI1 | -5.05 | 0.77 | 1.27 | 1.75 | 1.49 | up |  |
|  | Adipocyte plasma membrane-associated protein | Contig25844 | 9.16E-80 | 80 | EKC42337 | APMAP | 0.32 | 0.93 | 1.37 | 1.31 | 1.35 | up |  |
|  | Dipeptidyl-peptidase 1. partial | Contig34949 | 9.00E-120 | 64 | EKC33922 | DPP1 | -3.13 | -1.71 | -1.14 | -1.59 | -1.00 | down |  |
|  | Carboxypeptidase B | Contig35864 | 1.00E-78 | 53 | EKC34650 | CPB1 | -1.27 | 0.79 | 1.00 | 1.54 | 1.70 | up |  |
|  | Tetratricopeptide repeat protein 19 | Kmer2601832 | 2.19E-10 | 48 | EKC34342 | TTC19 | -1.35 | -0.12 | 1.33 | 1.38 | 1.39 | up |  |
|  | GnRH-related peptide precursor | Kmer2609810 | 2.66E-44 | 95 | BAH47639 | GNRH | -0.84 | 0.63 | 2.22 | 2.02 | 1.48 | up |  |
|  | Group XVI phospholipase A2 | Contig23068 | 2.30E-24 | 56 | EKC23544 | PLA2G16 | 0.50 | 1.51 | -6.19 | -1.27 | -1.58 | down |  |
|  | serine protease CFSP3 | Contig31517 | 2.65E-62 | 58 | ABB89132 | CFSP3 | -1.19 | -0.61 | -1.48 | -1.81 | -4.32 | down |  |
|  | predicted protein | Kmer2586618 | 1.82E-14 | 50 | XP_001623807 |  | -0.64 | 1.70 | -1.05 | -1.60 | -1.32 | down |  |
|  | carbonic anhydrase. putative | Kmer2613940 | 1.02E-10 | 46 | XP_002428435 | CA2 | -1.16 | -0.50 | -1.04 | -1.22 | -3.62 | down |  |
|  | Serum paraoxonase/arylesterase 1 | Kmer2590048 | 3.90E-19 | 61 | EKC25343 | PON1 | 1.59 | -0.36 | 2.85 | 1.16 | 1.49 | up |  |
|  | Alpha-methylacyl-CoA racemase | Kmer2593398 | 5.99E-78 | 77 | EKC18886 | AMACR | 0.25 | -0.17 | 2.68 | 1.75 | 1.17 | up |  |
|  | Acyl-coenzyme A thioesterase 4 | Kmer2613834 | 2.57E-30 | 53 | EKC26987 | ACOT4 | -3.81 | 0.07 | 2.14 | 1.09 | 1.64 | up |  |
|  | group XVI phospholipase A1/A2-like. partial | Contig16720 | 5.00E-14 | 40 | XP_004085715 | PLA2G16 | -2.37 | 1.67 | 1.81 | 1.71 | 1.72 | up |  |
|  | Protein C20orf11. glucose induced degradation protein 8 | Contig9673 | 1.27E-107 | 92 | EKC24371 | GID8 | -1.48 | 1.31 | 1.16 | 1.41 | 1.31 | up |  |
|  |  |  |  |  |  |  |  |  |  |  |  |  |  |
|  | Heat shock 70 kDa protein 12A | Contig25894 | 1.38E-128 | 72 | EKC18507 | HSPA12A | -0.73 | 0.45 | 1.12 | 1.06 | 1.12 | up |  |
| Stress Response | stearoyl-CoA desaturase-1 | Contig28270 | 4.49E-19 | 72 | AET74083 | SCD | -0.80 | -0.40 | -1.17 | -1.07 | -1.54 | down |  |
|  | Laccase-1 | Contig26352 | 3.40E-80 | 64 | EKC25936 | LAC1 | -1.06 | -1.30 | -3.37 | -2.26 | -3.58 | down |  |
|  | hypothetical protein BRAFLDRAFT_67911 | Contig31568 | 4.59E-138 | 75 | XP_002595129 |  | -0.21 | 0.86 | 1.75 | 1.40 | 1.04 | up |  |
|  | Neutral and basic amino acid transport protein rBAT | Contig32431 | 1.10E-110 | 63 | EKC19380 | RBAT | -0.51 | 0.32 | 1.83 | 1.38 | 1.02 | up |  |
|  | Laccase-1 | Contig8350 | 6.31E-100 | 61 | EKC25936 | LAC1 | -2.39 | -1.86 | -1.09 | -1.50 | -1.80 | down |  |
|  | hypothetical protein CAPTEDRAFT_220209 (immunophilin) | Contig9638 | 3.54E-47 | 85 | ELT92262 | FKBP45 | -5.60 | 1.39 | 2.93 | 1.05 | 2.11 | up |  |
|  | glutathione S-transferase sigma 3 | Contig20068 | 5.75E-32 | 66 | AFQ35985 | GSTS3 | -1.83 | -2.21 | -1.45 | -2.74 | -1.13 | down |  |
|  | calreticulin precursor | Kmer2615304 | 3.25E-177 | 91 | NP_001191523 | CALR | -2.08 | 1.50 | 1.13 | 1.67 | 2.31 | up |  |
|  | peptidoglycan recognition protein 4 | Contig10551 | 1.76E-40 | 73 | AAY27976 | PGLYRP4 | 0.58 | 4.05 | 3.99 | 4.44 | 3.87 | up |  |
|  | Steroid 17-alpha-hydroxylase/17.20 lyase | Contig28362 | 9.75E-67 | 66 | EKC22297 | CYP17A1 | -0.23 | 2.07 | 1.76 | 1.47 | 1.93 | up |  |
|  | heat shock protein 90 | Contig33569 | 0 | 99 | ABS50431 | HSP90 | -2.03 | 1.63 | 1.13 | 2.00 | 2.34 | up |  |
|  | hypothetical protein CAPTEDRAFT_183144 | Contig35018 | 1.50E-125 | 83 | ELT99118 |  | -1.53 | 1.29 | 1.65 | 1.06 | 1.37 | up |  |
|  | Major egg antigen | Contig28432 | 3.68E-122 | 72 | EKC31248 | P40 | -0.08 | 1.15 | 1.56 | 2.29 | 1.37 | up |  |
|  | Activator of 90 kDa heat shock protein ATPase-like protein 1 | Contig9726 | 1.24E-163 | 84 | EKC32134 | AHSA1 | -2.39 | 1.70 | 1.47 | 2.49 | 2.34 | up |  |
|  |  |  |  |  |  |  |  |  |  |  |  |  |  |
|  | Baculoviral IAP repeat-containing protein 7-A | Contig27457 | 1.84E-96 | 60 | EKC32616 | BIRC7 | -0.39 | 1.34 | 1.22 | 1.89 | 2.01 | up |  |
| Apoptosis related | Inositol 1.4.5-trisphosphate receptor type 1 | Kmer2589886 | 4.91E-59 | 77 | EKC35464 | ITPR1 | -0.35 | -0.47 | -1.20 | -1.82 | -1.32 | down |  |
|  | Baculoviral IAP repeat-containing protein 7-A | Contig36037 | 6.11E-86 | 73 | EKC32616 | BIRC7 | -1.29 | 1.84 | 2.39 | 1.81 | 1.53 | up |  |
|  | ariadne-1-like protein | Contig28903 | 1.02E-63 | 47 | EKC29163 | ARI1 | -2.74 | 1.14 | 1.60 | 1.85 | 3.35 | up |  |
|  | Tripartite motif-containing protein 2 | Contig34345 | 1.00E-65 | 51 | EKC26008. | TRIM2 | -1.41 | -0.35 | 1.06 | 2.51 | 3.14 | up |  |
|  | hypothetical protein | Kmer2620970 | 7.32E-17 | 43 | XP_002732174 |  | -0.47 | 0.97 | 2.11 | 1.63 | 3.88 | up |  |
|  | Baculoviral IAP repeat-containing protein 7-A | Contig18058 | 2.83E-80 | 63 | EKC38618 | BIRC7 | -1.30 | 1.68 | 2.04 | 2.00 | 1.30 | up |  |
|  | Ubiquitin carboxyl-terminal hydrolase 27 | Contig19723 | 1.42E-43 | 51 | EKC20536 | USP27X | -1.24 | 2.37 | 2.20 | 2.53 | 2.48 | up |  |
|  | Putative inhibitor of apoptosis | Contig26840 | 8.42E-56 | 58 | EKC17690 | PIAP | -0.77 | 2.40 | 2.15 | 1.88 | 1.60 | up |  |
|  | hypothetical protein CGI_10022970 | Contig35119 | 5.53E-12 | 46 | EKC38301 |  | -1.12 | 1.57 | 1.34 | 1.44 | 1.55 | up |  |
|  | Tripartite motif-containing protein 13 | Contig20284 | 5.75E-24 | 52 | EKC30865 | TRIM13 | -3.16 | 2.98 | 1.76 | 4.90 | 2.40 | up |  |
|  | Cell division cycle and apoptosis regulator protein 1 | Kmer2621852 | 1.40E-52 | 84 | EKC29909 | CCAR1 | -1.66 | 0.84 | 1.62 | 2.14 | 1.34 | up |  |
|  |  |  |  |  |  |  |  |  |  |  |  |  |  |
|  | Solute carrier family 43 member 3 | Contig33561 | 3.10E-18 | 48 | EKC31271 | SLC43A3 | 0.82 | -0.10 | 1.40 | 1.52 | 1.23 | up |  |
| Transport/ Homeostasis | Sodium-dependent phosphate transport protein 2B | Contig21791 | 0 | 74 | EKC36780 | SLC34A2 | 0.69 | 1.22 | 1.13 | 3.08 | 4.05 | up |  |
|  | uncharacterized protein LOC764526 isoform 1 | Kmer2596006 | 1.58E-39 | 62 | XP_001200862 |  | -0.23 | 0.85 | 1.03 | 1.37 | 3.75 | up |  |
|  | hypothetical protein CGI_10017460 | Contig25397 | 1.83E-20 | 60 | EKC30689 |  | 0.93 | 1.30 | 1.43 | 1.94 | 1.22 | up |  |
|  | annexin A7-like | Contig31351 | 6.37E-38 | 76 | XP_003383438 | ANXA7 | -1.88 | -1.53 | -1.52 | -1.35 | -2.09 | down |  |
|  | Protein disulfide-isomerase A6 | Contig32100 | 0 | 84 | EKC19533 | PDIA6 | -0.73 | 0.80 | 1.15 | 2.21 | 1.61 | up |  |
|  | Multidrug resistance-associated protein 1 | Contig35841 | 0 | 76 | EKC32378 | MRP1 | 1.50 | 6.07 | 3.49 | 7.99 | 2.36 | up |  |
|  | Sodium- and chloride-dependent glycine transporter 2 | Kmer2603250 | 3.46E-26 | 56 | EKC40069 | SLC6A5 | 0.26 | 0.08 | 1.93 | 2.50 | 1.58 | up |  |
|  |  |  |  |  |  |  |  |  |  |  |  |  |  |
| other | alpha macroglobulin | Contig9823 | 0 | 90 | AAR39412 | A2M | -1.63 | -0.18 | -1.24 | -1.57 | -1.10 | down |  |
|  | hypothetical protein LOTGIDRAFT_237271 | Kmer2613326 | 3.00E-22 | 35 | ESP04739 |  | -2.97 | -0.41 | -1.91 | -1.60 | -2.36 | down |  |
|  | hypothetical protein BRAFLDRAFT_171338 | Contig26581 | 6.91E-15 | 50 | XP_002600473 |  | -0.67 | 2.01 | 1.28 | 1.37 | 2.31 | up |  |
|  | Protein TSSC4 | Contig25750 | 1.17E-23 | 61 | EKC20638 | TSSC4 | -1.38 | 1.73 | 1.93 | 1.49 | 1.72 | up |  |
|  |  |  |  |  |  |  |  |  |  |  |  |  |  |
|  | NEFA-interacting nuclear protein NIP30 | Kmer2597016 | 3.83E-36 | 66 | EKC19734 | NIP30 | -2.09 | 1.22 | 1.60 | 1.16 | 1.91 | up |  |
|  | hypothetical protein CAPTEDRAFT_190177 | Contig34305 | 1.07E-11 | 54 | ELU07255 |  | 0.45 | 2.20 | 1.06 | 2.82 | 1.34 | up |  |
|  | Kelch-like protein 24 | Contig35538 | 1.54E-25 | 54 | EKC17247 | KLHL24 | -0.66 | 1.79 | 1.62 | 2.84 | 1.60 | up |  |
|  | UPF0420 protein C16orf58-like protein | Kmer2592130 | 1.55E-98 | 90 | EKC39300 | C16ORF58 | 0.32 | 1.63 | 1.12 | 2.77 | 1.24 | up |  |
|  | Kelch-like protein 24 | Contig16700 | 3.26E-27 | 50 | EKC17247 | KLHL24 | 0.32 | 2.71 | 2.35 | 3.34 | 2.83 | up |  |
|  |  |  |  |  |  |  |  |  |  |  |  |  |  |

**Table S2: List of genes found deregulated at 21°C. E-values, gene names and accession numbers are those given by Blast2GO for the best hit. Short names are, when possible, those of the best human homolog of the best hit (these names were used for String 9.05 analyses). Induction factors are given as Log2.**

| **Functional Classification** | **Gene Name** | **Contig ID** | **e-value** | **Id (%)** | **Acc** | **Short Name** | **3 d** | **14 d** | **21 d** | **27 d** | **56 d** | **21°C** | |
| --- | --- | --- | --- | --- | --- | --- | --- | --- | --- | --- | --- | --- | --- |
| Cytoskeleton/ Cell Binding | Titin | Contig29570 | 1.00E-62 | 39 | EKC32610.1 | TTN | -2.50 | 0.48 | 7.08 | 1.69 | 2.31 | up |  |
|  | Mesenchyme-specific cell surface glycoprotein | Contig23465 | 7.00E-72 | 48 | EKC20477.1 | MSP130 | 2.92 | -0.65 | 1.57 | 2.79 | 1.01 | up |  |
|  | Cartilage matrix protein | Contig30009 | 1.00E-45 | 30 | EKC20505.1 | MATN1 | -2.52 | -1.07 | 1.50 | 1.14 | 2.70 | up |  |
|  | Kelch-like protein 24 | Contig16700 | 5.00E-27 | 30 | EKC17247.1 |  | 2.21 | 1.02 | 1.07 | 2.87 | 1.33 | up |  |
|  |  |  |  |  |  |  |  |  |  |  |  |  |  |
| Replication / DNA Repair | pol-like protein | Kmer2621110 | 6.00E-53 | 27 | BAC82624.1 |  | 2.48 | 1.74 | 1.73 | 1.05 | 1.96 | up |  |
|  | Uracil-DNA glycosylase | Contig31738 | 4.00E-67 | 62 | EKC38003.1 | UDG | -2.75 | -1.87 | -2.58 | -2.30 | -1.25 | down |  |
|  | Uracil-DNA glycosylase | Contig22819 | 3.00E-81 | 58 | EKC38003.1 | UDG | -2.12 | -1.96 | -2.89 | -1.41 | -1.21 | down |  |
|  | Uracil-DNA glycosylase | Contig21363 | 3.00E-60 | 60 | EKC38003.1 | UDG | -5.74 | -1.66 | -4.08 | -2.92 | -1.16 | down |  |
|  | pol-like protein | Kmer2598858 | 1.00E-35 | 42 | BAC82626.1 |  | 2.81 | 1.18 | 2.33 | 1.38 | 4.26 | up |  |
|  | hypothetical protein | Contig34404 | 1.00E-44 | 32 | XP_003391328.1 |  | 1.86 | 0.97 | 2.77 | 1.67 | 3.04 | up |  |
|  | RNA-directed DNA polymerase from mobile element jockey-like | Contig1391 | 1.00E-34 | 52 | XP_003725849.1 |  | 1.21 | 3.25 | -1.36 | -3.38 | -1.09 | down |  |
|  | Uracil-DNA glycosylase | Contig32735 | 2.00E-70 | 53 | EKC38003.1 | UDG | -2.84 | 1.53 | -3.01 | -2.04 | -1.02 | down |  |
|  | transposase | Kmer2621654 | 1.00E-26 | 29 | AER39694.1 |  | -5.13 | 1.00 | -1.68 | -1.34 | -1.87 | down |  |
|  | Putative nuclease HARBI1 | Kmer2607486 | 2.00E-23 | 35 | EKC43123.1 | HARBI1 | -0.31 | 2.26 | -1.65 | -1.68 | -1.10 | down |  |
|  | uncharacterized protein | Contig34877 | 5.00E-40 | 53 | XP_004209790.1 |  | -2.01 | -1.85 | 1.75 | 1.39 | 1.17 | up |  |
|  | pol-like protein | Kmer2621110 | 6.00E-53 | 27 | BAC82624.1 |  | 2.48 | 1.74 | 1.73 | 1.05 | 1.96 | up |  |
|  | Uracil-DNA glycosylase | Contig31738 | 4.00E-67 | 62 | EKC38003.1 | UDG | -2.75 | -1.87 | -2.58 | -2.30 | -1.25 | down |  |
|  | Uracil-DNA glycosylase | Contig22819 | 3.00E-81 | 58 | EKC38003.1 | UDG | -2.12 | -1.96 | -2.89 | -1.41 | -1.21 | down |  |
|  |  |  |  |  |  |  |  |  |  |  |  |  |  |
| Protein modification/ maturation | hypothetical protein | Contig24181 | 2.00E-15 | 18 | XP_001619756.1 |  | 1.89 | 2.01 | 1.76 | 1.77 | 1.41 | up |  |
|  | VPS4A protein | Kmer2605336 | 9.00E-17 | 61 | AAH35121.1 | VPS4A | 0.92 | 0.73 | 2.61 | 1.08 | 1.04 | up |  |
| Signaling | TNF receptor-associated factor 3 | Contig30315 | 2.00E-53 | 54 | EKC42493.1 | TRAF3 | -0.66 | 1.32 | 1.15 | 2.55 | 1.81 | up |  |
|  | Prostaglandin reductase 1 | Contig25288 | 1.00E-61 | 57 | EKC24838.1 | PTGR1 | 0.27 | -0.82 | 1.44 | 1.43 | 1.06 | up |  |
|  | hypothetical protein | Kmer2589494 | 1.00E-57 | 69 | ESO84907.1 |  | 0.45 | -0.25 | 1.37 | 1.04 | 1.57 | up |  |
|  | Putative inhibitor of apoptosis | Contig12878 | 4.00E-57 | 31 | EKC26950.1 | XIAP | 0.34 | -0.45 | 1.25 | 1.14 | 1.28 | up |  |
|  | ariadne-1-like protein | Contig28903 | 1.02E-63 | 47 | EKC29163 |  | -0.78 | -0.21 | 1.58 | 1.92 | 1.30 | up |  |
|  | Sodium- and chloride-dependent glycine transporter 2 | Contig31254 | 4.00E-58 | 49 | EKC21522.1 | GST | 1.48 | 1.29 | 2.91 | 1.38 | 1.13 | up |  |
|  | peptidoglycan recognition protein 4 | Contig10551 | 3.00E-40 | 57 | AAY27976.1 | PGLYRP4 | 1.68 | 1.79 | 1.52 | 3.49 | 2.84 | up |  |
|  |  |  |  |  |  |  |  |  |  |  |  |  |  |
| other | hypothetical protein BRAFLDRAFT_122687 | Contig29522 | 2E-56 | 1 | XP_002591716.1 |  | -0.42 | 0.68 | 1.52 | 1.46 | 1.51 | up |  |
|  |  |  |  |  |  |  |  |  |  |  |  |  |  |
